# A Dysmorphic Child with a Pericentric Inversion of Chromosome 8

**DOI:** 10.1155/2012/813963

**Published:** 2012-02-08

**Authors:** Venkateshwari Ananthapur, Srilekha Avvari, Sujatha Madireddi, Pratibha Nallari, Jyothy Akka

**Affiliations:** ^1^Institute of Genetics and Hospital for Genetic Diseases, Osmania University, Begumpet, Hyderabad 500 016, India; ^2^Department of Genetics, Osmania University, Hyderabad 500 007, India

## Abstract

An 8-year-old boy was referred to our institute with dysmorphic features such as mild lupus, micrognathia, low hair line, hypoplasia, hemi atrophy of left side of the face, abnormal size of ears, hypothenar, hypoplasia of chin, and tongue tie. MRI scan was found to be normal and EEG suggestive of generalized seizure disorder. Cytogenetic evaluation of the proband revealed a pericentric inversion of chromosome 8 with 46, XY, and inv 8 (p11.2; q21.2) karyotype.

## 1. Introduction

Pericentric inversions are among the frequent chromosomal rearrangements associated with genetic disorders with a frequency of 1-2% [1, 2]. Pericentric inversions result from a two-break event which occurs between the short (p) and the long arms (q) within the chromosome followed by a 180° rotation of the intercalary segment. The phenotype of the inversion carrier depends on the type of inversion, size of the inverted part, and the chromosome involved [3]. In this report, we describe the distinct clinical phenotype and the karyotype of a boy with dysmorphic facial features and mild mental retardation associated with a pericentric inversion of chromosome 8.

## 2. Case Report

An 8-year-old male child with dysmorphic facies and mild mental retardation was referred to the Institute of Genetics, Hyderabad for cytogenetic evaluation. He was born after full term as the third child in the sibship of nonconsanguineous parents. He had delayed developmental milestones, neck holding at the age of 5 months, walking independently at the age of 2 years and 5 months, and started speech at the age of 3 years and 5 months. The dysmorphic facial features included mild lupeus, micrognathia, low-hair line, hypoplasia, hemiatrophy of left side of the face, abnormal size of ears, hypothenar, hypoplasia of chin, and tongue tie. His external genitalia were normal. Psychological evaluation of the child was carried out using Senguin form board and Vineland Social maturity physical examination scale [4]. The intelligent quotient was found to be 64 indicative of mild mental retardation.

MRI Scan report of the propositus was normal, but his EEG study was suggestive of generalized seizure disorder. He had hyperactive behavior with slurred speech. It is informed that the boy was frightened by loud sounds and is presently attending a special school.

**Figure 1:**
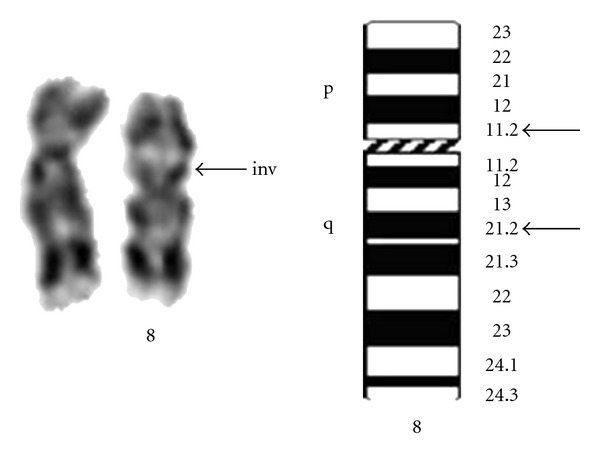
Partial karyotype of the propositus showing 46, XY, and inv 8 (p 11.2; q 21.2) and Ideogram of chromosome 8 with breakpoints.

Chromosomal analysis of peripheral blood lymphocytes was performed using GTG banding for the propositus and their parents [5, 6]. A rearranged chromosome was observed in the propositus with pericentric inversion of chromosome 8 with break point at p 11.2 and q 21.2 regions (Figure 1). The normal submetacentric chromosome 8 is seen as a metacentric chromosome after inversion. The parents showed normal chromosomal constitution there by indicating the chromosomal rearrangement in the proband as *de novo.*

## 3. Discussion

Inversion comprises approximately 10% of structural aberrations where pericentric inversions clearly outnumbered paracentric rearrangements. Pericentric inversion results from a two-break event in which there is a break in each arm including the centromere. Reorientation of a sequence of genetic material apparently does not influence its function, and breakage reunions at most sites do not cause an abnormal phenotype. Most pericentric inversions involve breakpoint in the centromeric heterochromatin of chromosome 1, 9, and 16. These inversions are generally inherited and considered clinically insignificant heteromorphisms as their breakpoints are in the repetitive noncoding sequences. In contrast, inversions with euchromatic breakpoint when they occur *de novo* are sometimes associated with abnormalities as there is a potential for gene disruption or position effect [7].

 Pericentric inversions have been described in every chromosome with varying frequencies. Inversions of chromosome 8 account for approximately 8% of all observed pericentric inversions, which include the rare occurrence of inversions in chromosome 1, 8, and 16. A possible reason for such rare finding may be due to unequal crossing over causing lethality [8].

Pericentric inversion of chromosome 8 has been reported in 50 unrelated families. The break points in the short arm involve band p 11, p 12, or p 2 while break points on the long arm are evenly distributed along all its length [9]. Stanley et al. [10] reported a case of recombinant 8 occurring in the offspring of a man carrying inv (8) (p 12; q 13) [9]. Another case of recombinant (8) was observed by Grix et al. [11] in a family where the chromosome abnormality was found to be inv (8) (p 23.2; q 23.1). Mattina et al. [9] reported inv (8) (p 23; q 22) in a Sicilian family which was also reported by others [9, 12]. In the present study, we report a novel pericentric inversion in the chromosome 8 with the novel break points of p 11.2 and q 21.2, in a dysmorphic child with mild mental retardation.

Some of the clinical features observed in the propositus are similar to the earlier reports of partial overlap of the chromosomal imbalance. However, the variable features such as mild mental retardation, facial dysmorphology, and hyperactiveness can be attributed to the inversion fragment between p 11.2 and q 21.2. [3, 11]. The phenotypic abnormality present in the child is suggestive of the involvement of euchromatin, wherein the break points occur in the active genes which result in the disruption of the gene/s function.

Analysis of the p 11.2 and q 21.2 bands at the genetic level showed the presence of genes; CHRNA2 gene-cholinergic receptor, nicotinic, alpha 2 (neuronal), FGFR1 gene-fibroblast growth factor receptor 1, GDAP1 gene-ganglioside-induced differentiation-associated protein and KCNQ3 gene-potassium voltage-gated channel, KQT-like subfamily, and member 3 which are essential for the normal functioning of the brain. Disruption of these genes due to the chromosome rearrangement may alter the modulation of the neuroproteins which in turn may influence normal functioning of brain resulting in mild mental retardation and seizures as seen in the propositus [13]. The rearrangement of the chromosome may thus result in altered gene function and expression of abrogated proteins which are implicated in gross delayed development resulting in dysmorphic features and mild mental retardation.

## References

[1] de la Chapelle A, Schroeder J, Stenstrand K (1974). Pericentric inversions of human chromosomes 9 and 10. *American Journal of Human Genetics*.

[2] Kaiser P (1984). Pericentric inversions. Problems and significance for clinical genetics. *Human Genetics*.

[3] Barnes ICS, Kumar D, Bell RJM (1985). A child with a recombinant of chromosome 8 inherited from her carrier mother. *Journal of Medical Genetics*.

[4] Kishore TMT, Nizamie SH, Nizamie A (1995). The behavioural profile of psychiatric disorders in persons with intellectual disability. *Journal of Intellectual Disability Research*.

[5] Moorhead PS, Nowell PC, Mellman WJ, Battips DM, Hungerford DA (1960). Chromosome preparations of leukocytes cultured from human peripheral blood. *Experimental Cell Research*.

[6] Seabright M (1971). A rapid banding technique for human chromosomes. *The Lancet*.

[7] Gardner RJM, Sutherland G (2004). Inversions. *Chromosomal Abnormalities and Genetic Counseling*.

[8] Borgoanker DS (1991). Chromosomal variation in man. *A Catalog of Chromosomal Variants and Anomalies*.

[9] Mattina T, Conti L, Milone G, Marino S, Sorge G (1989). Inv(8)(p23q22) and recombinant derivative in a Sicilian family. *Clinical Genetics*.

[10] Stanley RG, Dev GV, Butler MG, Philips JA (1989). Partial 8q trisomy and 8p monosomy resulting from inversion in paternal chromosome 8. *The American Journal of Human Genetics*.

[11] Grix S, Sherman S, Golabi M, Finbeiner W, Herva R, de la Chapelle A (1976). A large pericentric inversion of chromosome 8. *The American Journal of Human Genetics*.

[12] Moedjono SJ, Sparkes RS (1980). Familial pericentric inversion of chromosome 8; is breakpoint p23q23 important in the formation of unbalanced recombinants?. *Annales de Genetique*.

[13] Williams ThM, McConnell ThS, Martinez F, Smith ACM, Sujansky E (1984). Clinicopathologic and dysmorphic findings in recombinant chromosome 8 syndrome. *Human Pathology*.

